# Special Issue: “Molecular Signatures of Gynecological Cancers—Breast Cancer, Ovarian Cancer, Cervical Cancer, and Endometrial Cancer 2.0”

**DOI:** 10.3390/ijms252011275

**Published:** 2024-10-20

**Authors:** Lyudmila Gulyaeva

**Affiliations:** 1 Federal Research Center “Fundamental and Translational Medicine”, Timakov St. 2, 630060 Novosibirsk, Russia; lfgulyaeva@gmail.com; 2 Department of Clinical Biochemistry, Zelman Faculty of Medicine and Psychology, “Novosibirsk State University”, Pirogov St. 1, 630090 Novosibirsk, Russia

Cancer of women’s reproductive organs (gynecological cancers) and breast cancer affect women all over the world. Scientific and medical research data indicate that the frequency as well as mortality rate of such cancers are continuously rising. Therefore, improving patient survival requires further development of diagnostic and therapeutic approaches. Significant progress has been made in targeted therapy for gynecological cancers based on emerging knowledge of the molecular and genetic characteristics of tumors. In particular, individualized therapy based on patient tumor characteristics markedly improved the therapeutic outcomes and patient survival. This is particularly evident in cases with high MSI (for example, for endometrial cancer), where the immune checkpoint inhibitors dostarlimab and pembrolizumab/lenvatinib are the most effective [1]. HRD detection in high-grade ovarian carcinoma as an indicator for using PARP inhibitors gave promising results [2]. However, there are still many unresolved therapeutic challenges, including triple-negative breast cancer, especially at an advanced stage.

This Special Issue consists of nine original articles and two reviews that focus on studying the mechanisms of carcinogenesis as well as clinical aspects, including a search for novel markers for prognosis and treatment. The article by Anastasia Maltseva et al. focuses on immunotherapy in endometrial cancer and shows the important role of the tumor microenvironment [3]. The authors make an important statement that the proportion of CD20+ B lymphocytes and the CD8-to-CD20 lymphocyte ratio in the stroma of endometrial cancer serves as both a prognostic marker of response to immunotargeted therapy and a prognostic factor for progression-free survival in patients. Rong Bu et al. studied Lynch syndrome (LS) in patients with endometrial cancer and showed for the first time that 12.2% of patients in Saudi Arabia were DNA mismatch repair-deficient (dMMR) [4]. The authors performed a thorough analysis of damage using IHC to detect dMMR, an MLH1 promoter hypermethylation analysis, as well as a whole-exome sequencing analysis.

Seven cases were found to have varied and uncertain significance in cancer-related genes other than the MMR genes. This study is important for identifying women with LS and LLS in order to form risk groups, as well as to use immunotherapy for patients with endometrial cancer. Several studies aimed to identify novel markers for breast cancer: It was shown early that ASAP1, also known as ANK repeat and PH domain-containing protein 1, is associated with poor prognosis in various types of cancer, including hepatocellular carcinoma and gastric cancer [5,6]. Park et al. showed that low ASAP1 expression was associated with worse recurrence-free survival in invasive breast cancer, especially in ER-positive cases [7]. Furthermore, Li et al. [8] studied the mechanism of action for the long non-coding RNA (lncRNA) actin fiber-associated protein-1 antisense RNA 1 (AFAP1-AS1) and showed that it exerts oncogenic activity in triple-negative breast cancer (TNBC). The authors conclude that the AFAP1-AS1 upstream interacts with the transcription factor SP1 and modulates the mTOR signaling downstream, thus offering insights into the tumorigenic mechanism in TNBC. Besides AFAP1-AS1, another potential marker of TNBC is described—ankyrin repeat domain containing 11 (ANKRD11/ANCO1) [9], the low expression of which was observed in some cancers.

The authors have shown that low ANCO1 mRNA and protein expression levels are prognostic markers for poor clinical outcomes in breast cancer and that a loss of nuclear ANCO1 protein expression predicts a lower overall survival of patients with TNBC. Notably, the ChIP-seq analysis in ANCO1-depleted cells shows a global increase in H3K27Ac in regions that are enriched for the AP-1, TEAD, STAT3, and NFκB motifs. Special attention is paid to the tumor microenvironment in breast cancer (for example, see the comprehensive review by Kotsifaki et al.) with a description of the potential biomarkers associated with the immune response [10]. In addition to the immune cells associated with the tumor, cytokine expression in the breast cancer microenvironment, immune evasion in breast cancer, and the role of immune checkpoints were analyzed. This review is very useful for understanding the tumor response to immunotherapy, which does not so far show very promising results in breast cancer. The second review discusses the latest knowledge on the inhibitor of metalloproteases (MMPs), TIMP3, that is associated with cervical adenocarcinoma as well as progressed ovarian cancer and breast cancer metastasis [11]. The authors note that the relationship between TIMP3 and endometrial cancers remains unclear. However, TIMP3 may still be a useful biomarker for gynecological cancers and is a potential target for future cancer therapy.

Several articles are dedicated to ovarian cancer (OC), one of the most malignant and aggressive gynecological cancers that remains to be very challenging to diagnose at early stages. The article by Yunusova et al. [12] provides an excellent study of exosomes that could be involved in the development, migration, and invasion of cancer cells and the cargo of which could be useful for non-invasive biopsy development. The authors show that the levels of tetraspanins Tspan8+ and CD151+ exosomes are significantly higher in the plasma of OC patients compared to healthy females (HFs). The authors also show that the relative levels of miR-24 and miR-101 in the plasma exosomes of HFs are significantly higher than those in the plasma exosomes of OC patients. Undoubtedly, these results are instrumental for an early diagnosis of ovarian cancer as well as treatment monitoring. One of the most promising treatments for ovarian cancer are those involving poly ADP–ribose polymerase (PARP) inhibitors for tumors with homologous recombination deficiency (HRD). According to The Cancer Genome Atlas, an HRD-positive status is the most prevalent alteration in ovarian cancer (69%). In the article by Kekeeva et al. [13], the methods that are routinely used in the clinic to determine an HRD phenotype, including telomeric allelic imbalance, large-scale transition, and loss of heterozygosity, are analyzed. The authors determine the HRD status, including *BRCA* status and genomic scar score (GSS), in 452 ovarian cancer specimens using the AmoyDx HRD Focus Panel for high-grade serous ovarian cancer. The rate of positive HRD testing was 86%. Thus, the researchers conclude that the HRD rate in the Russian population is very similar to those of other European populations, as is the *BRCA* mutation frequency. This article can be particularly useful for physician oncologists to assess the effectiveness of platinum drugs and PARP inhibitors used for the treatment of OC. The article by Scebba et al. [14] looks into OC biomarkers for early diagnosis. The authors compare the saliva samples of patients with various stages of OC or breast cancer (BC) and those of healthy subjects using an unbiased, high-throughput proteomics approach. Importantly, they show that saliva can be an informative test fluid for an unbiased proteomic search for candidate biomarkers, notably RAD51 isoform 2, Serum amyloid A (SAA) 1 protein, the truncated form of Apolipoprotein (APO) A2, and macrophage migration inhibitory factor (MIF). Further development of this approach with clinical verification of the markers will help establish non-invasive methods for early OC detection.

One study investigated the mechanism of metastasis of cervical cancer, also one of the most malignant gynecological cancers [15]. The authors hypothesized that PenToXifylline (PTX), which possesses potent anti-inflammatory effects, can exert anti-EMT (epithelial–mesenchymal transition) effects. PTX is a methylxanthine derivative that inhibits phosphodiesterases. Due to its anti-inflammatory effect, PTX is used in the treatment of a variety of diseases and pathologies, including those of dermatological, hemorheological, and cardiac nature, as well as COVID-19.

The authors obtained promising results that show that PTX inhibits EMT in cervical cancer cells by decreasing the NF-κB and SERPINE1.

Overall, the eleven articles featured in this Special Issue will have a significant impact on the future development of novel methods for the diagnostics, treatment, and prognosis of gynecological cancers.
